# Brief Report: Intravital Imaging of Cancer Stem Cell Plasticity in Mammary Tumors

**DOI:** 10.1002/stem.1296

**Published:** 2012-12-07

**Authors:** Anoek Zomer, Saskia Inge Johanna Ellenbroek, Laila Ritsma, Evelyne Beerling, Nienke Vrisekoop, Jacco Van Rheenen

**Affiliations:** Hubrecht Institute-KNAW and University Medical Center UtrechtUtrecht, The Netherlands

**Keywords:** Cancer stem cells, Intravital lineage tracing, Mammary imaging window, Breast cancer, MMTV-PyMT

## Abstract

It is widely debated whether all tumor cells in mammary tumors have the same potential to propagate and maintain tumor growth or whether there is a hierarchical organization. Evidence for the latter theory is mainly based on the ability or failure of transplanted tumor cells to produce detectable tumors in mice with compromised immune systems; however, this assay has lately been disputed to accurately reflect cell behavior in unperturbed tumors. Lineage tracing experiments have recently shown the existence of a small population of cells, referred to as cancer stem cells (CSCs), that maintains and provides growth of squamous skin tumors and intestinal adenomas. However, the lineage tracing techniques used in these studies provide static images and lack the ability to study whether stem cell properties can be obtained or lost, a process referred to as stem cell plasticity. Here, by intravital lineage tracing, we report for the first time the existence of CSCs in unperturbed mammary tumors and demonstrate CSC plasticity. Our data indicate that existing CSCs disappear and new CSCs form during mammary tumor growth, illustrating the dynamic nature of these cells. Stem Cells *2013;31:602–606*

## INTRODUCTION

Tumors are heterogeneous and contain multiple cell types. It has been widely hypothesized that, similar to epithelial tissues, mammary tumors are hierarchically organized where only a small number of stem-like cells referred to as cancer stem cells (CSCs) is able to maintain and provide tumor growth [1–3]. Until recently, evidence for the presence of CSCs in primary tumors came from transplantation assays, which study the capacity of specific tumor cell subpopulations to reform a tumor identical to the parental tumor upon transplantation into immunodeficient mice. Although these studies clearly demonstrate the clonogenic capacity of specific cancer cells under transplantation conditions, it has been widely disputed whether this assay accurately reflects cell behavior required for growth of an unperturbed tumor [1–3]. To overcome this technical limitation, lineage tracing techniques have been used to study the hierarchical organization of squamous skin tumors [4] and intestinal adenomas [5]. In these studies, CSCs are defined as the population of cells that provide tracing and therefore are able to maintain and provide tumor growth [4, 5]. Nevertheless, it has been speculated that cells only temporally adapt these stem cell properties [6–10]. The gain or loss of these properties, which we refer to as stem cell plasticity, include non-CSCs that (temporally) become CSCs, quiescent CSCs that become active, CSCs that lose stemness, and CSCs that become quiescent [10–13]. Lineage tracing experiments as performed in the above-mentioned studies allow the study of the hierarchical organization of tumors; however, they lack the ability to test the CSC plasticity hypothesis, since they provide static images. Here, we investigate whether the growth of mammary tumors is hierarchically organized and whether this organization is dynamic using a genetic mouse model and intravital microscopy techniques. In addition to the recent identification of CSCs in squamous skin tumors [4] and intestinal tumors [5], we report the presence of mammary CSCs in nonmanipulated primary mammary tumors and provide the first experimental evidence for the existence of CSC plasticity.

## MATERIALS AND METHODS

### Mice

MMTV-PyMT mice were purchased from Jackson Laboratory, Sacramento, CA, jaxmice.jax.org. R26-CreERT2 and R26-Confetti mice were kind gifts from the labs of Dr. Jacqueline Deschamps and Dr. Hans Clevers, respectively. All mice were housed under standard laboratory conditions and received food and water ad libitum. Five milligrams of tamoxifen (Sigma, St. Louis, MO, http://www.sigmaaldrich.com) dissolved in regular sunflower oil was injected intraperitoneally in mice bearing PyMT adenomas (mice 5 weeks of age) or carcinomas (mice 13 weeks of age); tumor progression stages were determined by histological analyses (Supporting Information Fig. S1). All experiments were carried out in accordance with the guidelines of the Animal Welfare Committee of the Royal Netherlands Academy of Arts and Sciences, The Netherlands.

### Mammary Imaging Window Surgery

To intravitally trace the growth of PyMT tumors over time, a mammary imaging window was inserted. Mice were anesthetized using 2% isoflurane/medical air anesthesia. Surgical procedures were performed under aseptic conditions. Before surgery, the skin overlying the tumor was shaved and depilated, and the skin was disinfected using 70% ethanol. An incision was made through the skin overlying the tumor and an imaging window was inserted (for details see [15, 17, 18]). The imaging window was secured using a nonabsorbable nonwoven purse-string suture (4-0 prolene suture). At the end of the surgical procedure, 5 mg tamoxifen was injected intraperitoneally to activate the confetti color randomizer.

### Intravital Imaging

Mice were anesthetized using isoflurane inhalation (1.5%–2% isoflurane/medical air mixture) and placed in a facemask within a custom designed imaging box. Isoflurane was introduced through the facemask and ventilated by an outlet on the other side of the box. The imaging box and microscope were kept at 32°C by a climate chamber surrounding the entire stage of the microscope including the objectives. Imaging was performed on an inverted Leica TCS SP5 AOBS multiphoton microscope (Mannheim, Germany, Leica-microsystems.com) with a chameleon Ti:Sapphire pumped Optical Parametric Oscillator (Coherent Inc., Santa Clare, CA, http://www.coherent.com). The microscope is equipped with four nondescanned detectors: NDD1 (<455 nm), NDD2 (455–490 nm), NDD3 (500–550 nm), and NDD4 (560–650 nm). Different wavelengths between 700 nm and 1,150 nm were used for excitation; CFP was excited with a wavelength of 840 nm, and GFP, YFP, and RFP were excited with a wavelength of 960 nm. CFP and GFP were detected in NDD2, YFP was detected in NDD3, and RFP in NDD4. All images were in 12 bit and acquired with a 25× (HCX IRAPO N.A. 0.95 WD 2.5 mm) water objective.

### Tracing Dynamics of Hierarchical PyMT Carcinoma Growth

A mammary imaging window was surgically implanted onto PyMT carcinomas, and the hierarchical tumor growth was intravitally imaged over the following weeks through the window. Three-dimensional tile scans of large tumor areas (at least 3 × 3 × 0.3 mm^3^ [*XYZ*] with 10 μm *Z* steps) were made at indicated time points. Typically, three to seven tile scans were collected over time periods of up to 3 weeks after induction of the confetti color randomizer. Imaging coordinates of the first imaging session were saved using Leica Application Suite AF software and used for subsequent imaging sessions to retrace the same imaging fields. All intravital images were processed using custom-made software (codes on request available from J.v.R.) and ImageJ software (NIH, Bethesda, MD, http://www.nih.gov). Custom-made software was used to crop regions of interest from the large three-dimensional tile scans, and ImageJ software was used to convert 12-bit images from the five channels into a single RGB image and to generate maximum projections of the three-dimensional volumes (100–200 μm deep). If necessary, images were smoothed and contrasted linearly.

Clone sizes were measured using the freehand selection tool in ImageJ and divided into the following growth patterns: continuous growth (>25% size increase compared to earlier time points), disappearance, regression (>25% size decrease compared to earlier time points), and/or a delayed onset of growth (clone which is undetectable at earlier time points but can be detected in later intravital imaging sessions). Four mice were analyzed to determine PyMT clone growth patterns.

### Immunohistochemistry

Tumors were isolated, fixed in 4% Paraformaldehyde, paraffin-embedded, and sectioned (4-μm thick) with a Leica microtome RM2235 (Mannheim, Germany, http://www.leicabiosystems.com). Sections were stained for hematoxylin and eosin, smooth muscle actin (mouse monoclonal antibody; 1:2,000; Sigma, St. Louis, MO, http://www.sigmaaldrich.com), Ki67 (mouse monoclonal antibody; 1:300; Monosan, Uden, The Netherlands, http://www.monosan.com), or cleaved caspase-3 (rabbit monoclonal antibody;1:400; Cell Signaling Technology, Boston, MA, http://www.cellsignal.com) according to standard histological protocols. Anti-mouse Envision-PO antibody (Dako, Glostrup, Denmark, http://www.dako.com) or anti-rabbit HRP antibody (Immulogic, Woburn, MA) were used as secondary steps. Images were acquired using a Nikon Eclipse E600 microscope (Nikon, Tokyo, Japan, http://www.nikoninstruments.com).

### Statistics

A Student's *t* test using Microsoft Excel (Redmond, WA, http://www.microsoft.com) was used to determine statistical significant differences between two means. A two-way ANOVA test using GraphPad Prism (GraphPad Software, LA Jolla, CA, http://www.graphpad.com) was used to determine statistical significant differences in tumor growth between two groups. Differences were considered statistical significant when *p* < 0.05.

## RESULTS

We use a genetic mouse model based on the overexpression of Polyomavirus middle T antigen (PyMT), which recapitulates human breast tumor progression and morphology [14]. As reported before [14], PyMT mice first develop adenomas in which the myoepithelial basement membrane is present (Supporting Information Fig. S1, left panel), which progress to carcinomas that lack the basement membrane, and contain invasive fronts where tumor cells invade the stroma (asterisk in Supporting Information Fig. S1, right panel).

**Figure 1:**
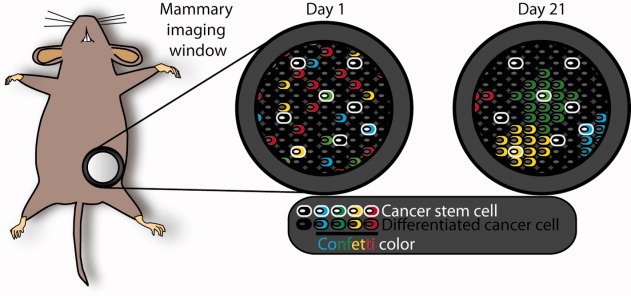
Intravital lineage tracing shows cancer stem cell plasticity in mammary tumors. Cartoon of the experimental setup. The four confetti colors (cyan fluorescent protein, green fluorescent protein, yellow fluorescent protein, and red fluorescent protein) are randomly expressed in mice bearing genetic mammary tumors. By multiple intravital imaging sessions through a mammary imaging window, the progeny (which inherits the expression of the fluorescent proteins) of each cancer stem cell (CSC) can be traced. By stochastically inducing confetti colors in both non-CSCs and CSCs, plasticity of CSC properties can be visualized without excluding subtypes or differentiated cells that do not (permanently) express stem cell markers such as quiescent CSCs or non-CSCs that become CSCs.

To study whether mammary tumor growth is hierarchically organized, we developed intravital lineage tracing tools to trace individual tumor cells and their progeny in the same animal over periods of up to 3 weeks (Fig. 1). Through our mammary imaging window (Fig. 1 and [15]), we lineage traced the growth of genetic mammary tumors that in addition to PyMT also express a Cre-inducible confetti construct. In these mice, Cre activity induces expression of one of four confetti colors (cyan fluorescent protein [CFP], green fluorescent protein [GFP], yellow fluorescent protein [YFP], and red fluorescent protein [RFP]) [16]. Therefore, Cre-expressing cells and their progeny will be genetically marked by one of the confetti colors, which allows intravital lineage tracing of these cells. We have chosen to drive expression of tamoxifen-inducible Cre (CreERT2) by the ubiquitous promoter ROSA26 (R26)—and not a stem cell promoter—since to date no definitive markers have been identified that specifically label CSCs in mammary tumors. Although tamoxifen stochastically induces expression of one of the four confetti colors in both stem cells and differentiated cells, only cells with stem cell properties will maintain tumor growth and result in tracing over time. Importantly, this labeling approach includes cells that do not express established stem cell markers, including currently unidentified subtypes of CSCs, quiescent CSCs, and non-CSCs that become CSCs.

**Figure 2:**
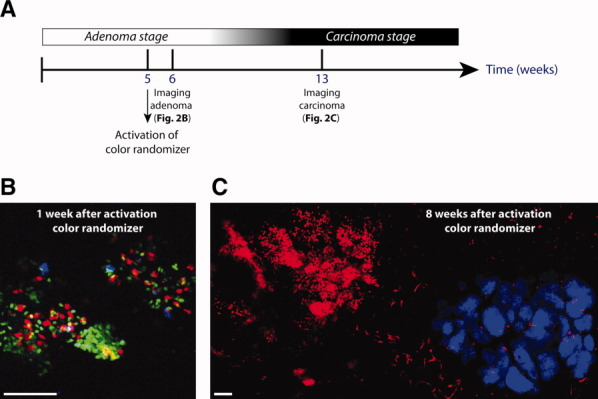
Lineage tracing of mammary adenoma growth. **(A):** Cartoon depicting the timeline of the experiment where expression of the confetti colors was induced in the adenoma stage and images were acquired at the adenoma and carcinoma stages. **(B):** Image of an adenoma 1 week after induction of the confetti randomizer. **(C):** Image of a carcinoma 8 weeks after induction of the confetti randomizer. In all images, blue: CFP^+^ cells, green: YFP^+^ cells, red: RFP^+^ cells. Scale bars = 100 μm.

**Figure 3:**
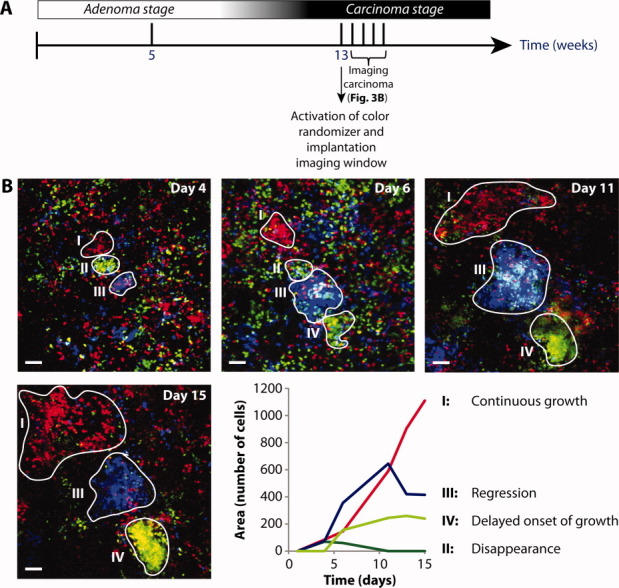
Imaging organizational hierarchy in mammary carcinoma growth. **(A):** Cartoon depicting the timeline of the experiment where expression of the confetti colors was induced in the carcinoma stage and the hierarchical organization of carcinoma growth was intravitally imaged over time through an imaging window. **(B):** Series of intravital images of a growing carcinoma at the indicated time points after induction of the confetti randomizer. Different growth patterns of clones were observed (indicated by I–IV). Region I displays continuous growth, regions II and III show growth before disappearance or regression, respectively, and region IV shows a delayed onset of clonal outgrowth. All different growth patterns have been observed at least 10 times in four mice. In all images, blue: CFP^+^ cells, green: YFP^+^ cells, red: RFP^+^ cells. White lines indicate the outlines of the differently colored clones in the graph. Scale bars = 50 μm.

At 5 weeks of age, mice developed breast adenomas consisting of millions of cells. At this stage, we turned on the confetti color randomizer by tamoxifen administration (Fig. 2A) which led to the stochastic expression of confetti colors (Fig. 2B), but did not result in detectable changes in tumor morphology, proliferation, apoptosis, and tumor growth (Supporting Information Fig. S2). At the carcinoma stage, large lobes consisted of a single confetti color (Fig. 2C), demonstrating that of all cells in the adenoma stage, only a fraction of tumor cells clonally expanded. To test whether the founder CSC of a clonal lobe gives rise to differentiated cancer cells only or additionally forms new CSCs, we induced the confetti color randomizer in carcinomas (Fig. 3A). After tamoxifen administration, we inserted an imaging window to lineage trace the growth of carcinomas over a period of up to 3 weeks. Interestingly, we observed multiple single-colored patches of different sizes throughout the clonal carcinoma lobes (Fig. 3B). These data imply the formation of new CSCs from the lobe-founding CSC. In line with these results, we observed that CSC properties were occasionally acquired and/or activated days after color-induction, resulting in a delayed onset of tracing (Fig. 3B). We also observed the regression and disappearance of patches, indicating the loss of stem cell properties (Fig. 3B).

## DISCUSSION AND CONCLUSIONS

Using intravital lineage tracing in a genetic mouse model for breast cancer we have shown for the first time the existence of CSCs in unperturbed mammary tumors. By activation of a color randomizer, which allows tracing of lineages by the induction of confetti colors, at different stages of tumor development we have shown that only a small fraction of tumor cells clonally expands. The few cells with stem cell capacities provide tumor growth by dividing into differentiated tumor cells but they also give rise to new cells with stem cell capacities, again able to clonally expand. These results indicate that the stem cell state is plastic, and can be acquired, lost and/or (de-)activated. Since CSCs can disappear, loss of a CSC may be compensated by the proliferation of the neighboring CSC leading to neutral drift. Whether this explains the clonality of the tumor tissue as shown in Fig. 2C is currently under investigation. Moreover, further research will elucidate whether our results hold true for breast tumors only or whether plasticity represents a more common property of CSCs.

## Supplementary material

Supporting Information Figure S1. PyMT expression induced the formation of adenomas that progressed to carcinomas. Shown are two histological images of sections of a PyMT adenoma (left) and a PyMT carcinoma (right) stained for hematoxylin (purple) and smooth muscle actin (brown). Note that in the adenoma the smooth muscle actin-positive basement membrane is still present, but is lacking in the carcinoma. Moreover, tumor cells in the carcinoma invade the surrounding stroma (marked by the asterisk). Scale bars, 50 μm.

Supporting Information Figure S2. PyMT tumor growth was not detectable affected by the induction of the confetti randomizer using tamoxifen. (A): Shown are histological images of sections of a PyMT tumor treated or not treated with tamoxifen stained for hematoxylin and eosin. Scale bars, 50 μm. (B): Other PyMT sections of the same tumors as in A were stained with an anti-Ki67 antibody, and the number of proliferative cells per imaging field (i.f.) was analyzed (N=25). The average number and standard error of the mean of proliferative Ki67+ cells were plotted. (C): Other sections were stained for apoptotic cells with an anti cleaved caspase-3 antibody. The average number and standard error of the mean of apoptotic cells per imaging field (N=25) were plotted. Shown are the average number and standard error of the mean. (D): Growth of PyMT tumors in control (gray line) and tamoxifen-treated animals (red line) plotted over time. Shown are the average values and standard error of the mean for 4 mice.
