# Supplementary figures and images for: Brief Report: Intravital Imaging of Cancer Stem Cell Plasticity in Mammary Tumors
Source: Stem Cells. 2012 Dec 7;31(3):602–6. doi: 10.1002/stem.1296 (PMC3744756; doi:10.1002/stem.1296)

Adenoma

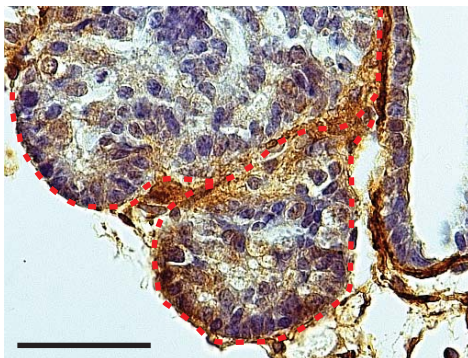

Carcinoma

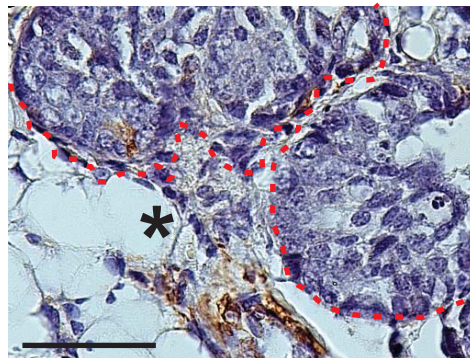

Smooth muscle actin / Hematoxylin

Supplement: Supplementary file 1 [file stem0031-0602-SD1.pdf]

**A**

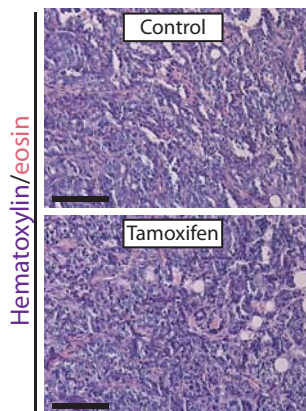

**B**

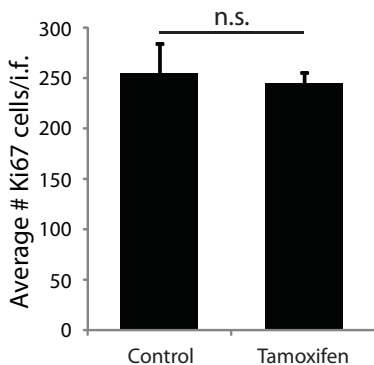

**C**

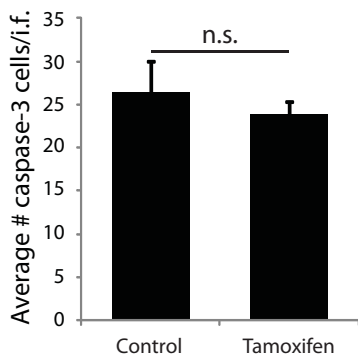

**D**

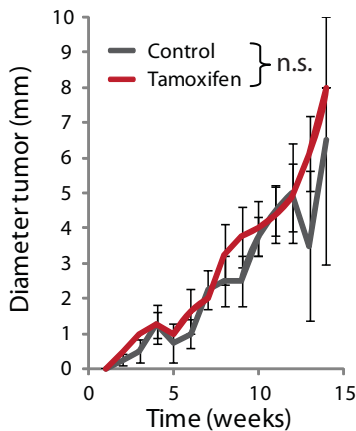

Supplement: Supplementary file 2 [file stem0031-0602-SD2.pdf]
